# Ultrasound assisted magnetic dispersive solid phase microextraction for Hg determination in fuel oils using inductively coupled plasma optical emission spectroscopy

**DOI:** 10.1038/s41598-025-01447-8

**Published:** 2025-05-13

**Authors:** Mxolisi J. Kiwanuka, Philiswa N. Nomngongo, Nomvano Mketo

**Affiliations:** 1 Department of Chemistry, College of Science, Engineering and Technology (CSET), University of South Africa, Florida Science Campus, Johannesburg, 1709 South Africa; 2 Department of Chemical Sciences, Faculty of Science, University of Johannesburg, Johannesburg, South Africa

**Keywords:** Chemistry, Materials science, Nanoscience and technology

## Abstract

Mercury is considered a global pollutant, and its quantification in fuel oils remains a priority for public health and the ecosystem. Functionalized graphene oxide has gained popularity for heavy metal adsorption in various matrices. Herein we present a highly selective and efficient magnetic graphene oxide coated with gold (Fe_3_O_4_/GO-Au) nano adsorbent and its application to a dispersive solid-phase microextraction of mercury in fuel oils. The synthesized Fe_3_O_4_/GO-Au nanocomposite was characterized using Fourier Transform Infra-Red spectroscopy (FT-IR), powder X-ray diffraction (P-XRD), Scanning electron microscopy (SEM/EDS), Transmission electron microscopy (TEM), Thermogravimetric (TGA), Brunauer-Emmett-Teller (BET), and vibration sample magnetometer (VSM) analysis. The multivariate optimum conditions for the extraction method were 20 min, 30 mg, pH 7, and 1.75 mol/L, for adsorption time, sorbent mass, pH, and eluent concentration, respectively. Under the optimized conditions, the dynamic linear range of 0.1 to 100 µg/L (R^2^ = 0.999) was obtained. The limit of detection (LOD) and preconcentration factor obtained were 0.035 µg/L and 255, respectively. Intra- and inter-day precision of the method were calculated to be 3.5% and 4.0%, respectively. The newly developed method was successfully applied in real crude oil, diesel oil, gasoline, and kerosene samples. The environmental impact of the newly developed procedure was evaluated and a total score of 0.64 was obtained.

**Supplementary Information:**

The online version contains supplementary material available at 10.1038/s41598-025-01447-8.

## Introduction

Mercury is a hazardous contaminant found in various environmental media such as sediments, wastewater, soil, and natural water bodies. The former also occurs naturally in energy sources such as crude oil. The latter is exposed to mercury due to the nature of its formation over a long period^1^. The processing and burning of fuel oils release mercury into the atmosphere which result to environmental pollution. Mercury is a toxic element even at very low concentrations^2^. Mercury species are associated with detrimental health effects such as changes in DNA structures, damage to the liver, kidneys, digestive system, immune and nervous system^3^. There are various analytical techniques which are employed for the analysis of mercury. These include cold vapor atomic absorption spectroscopy (CV-AAS), inductively coupled plasma optical emission spectrometry (ICP-OES), inductively coupled plasma mass spectrometry (ICP-MS), atomic fluorescence spectrometry (AFS)^4^ among others. However, due to very low concentrations of mercury and high organic content of fuel oils a sample preparation step is necessary to separate and preconcentrate the analyte prior to its analytical analysis^5^.

Among other preconcentration techniques, magnetic solid phase extraction (MSPE) has proven to be a versatile technology that can be employed in different samples. The MSPE procedure allows sorbent to be dispersed within a large number of samples and hence facilitates its separation by using an external magnetic field^6,7^. In MSPE the choice of the adsorbent is a critical factor to obtain full recovery and high selectivity^8^. The advantages of MSPE include low costs, rapidity, simplicity, and reusability. Metal oxides have received wide attention in extraction for the preconcentration of metal ions. Magnetite (Fe_3_O_4_) is the most utilized transition metal oxide with various industrial applications such corrosion control, heterogeneous catalysis and magnetic storage of information^9^. The former have lot of advantages such as structural changes that allow the altering of lattice symmetry and cell parameters, high thermal and mechanical stability, low costs, biocompatibility, high saturation of magnetism and less toxicity^10–12^.Nevertheless, pure magnetic nanoparticles tend to agglomerates, as well as they are chemically active and oxidized in air, resulting in loss of magnetism^13^.

Graphene oxide has been investigated as the perfect material to avoid agglomeration of Fe_3_O_4_ nanoparticles. The former is a good sorbent because of its two-dimensional geometrical structure and the availability of hydroxyl, epoxy, and carboxyl groups on its surface. These oxygen-containing functional groups can exchange a single pair of electrons with those at their basal plane and edges, thus attaching heavy metal ions efficiently^14^. Nevertheless, the adsorption capacity of GO for Hg (II) is, however, negligible, or non-existent. To circumvent this challenge, gold nanoparticles are preferred because surface Au has a high affinity for mercury and forms Au-Hg amalgams^15^. Gold ions (Au^3+^) were reduced to gold atoms (Au^0^) by a reducing agent on the surface of the magnetic graphene oxide. The ability of gold to adsorb and amalgamate Hg has been widely reported in literature. However, the reaction depends on sizes and shapes^16^. In 2012, Dong-Hee Lim et al.^17^ investigated the adsorption behaviour of Hg onto Au using density fractional theory (DFT). M. Levlin and coworkers^18^ investigated the adsorption of Hg onto gold surfaces with a scanning tunnelling microscopy (STM). Their results showed that the adsorption included place exchange processes and concerted adsorption of more than one Hg atom in one process. Battistoni and coworkers^19^ echoed an interesting study for the adsorption of Hg on Au surfaces. Their results were confirmed using X-ray photoelectron spectroscopy (XPS), small area X-ray photoelectron spectroscopy (SAXPS), secondary ion mass spectroscopy (SIMS), scanning Auger microscopy (SAM) and scanning electron microscopy (SEM). Their SIMS and SAXPS results suggested that mercury is absorbed into a thin surface sublayer.

In this study, Fe_3_O_4_/GO-Au nanocomposite sorbent was synthesized by a simple method to extract mercury from fuel oils. To the best of our knowledge, it is the first time the Fe_3_O_4_/GO-Au nanocomposite was investigated for the preconcentration of total mercury in fuel samples. The mixture of HCl and thiourea was used as an eluent and the extracts were taken for analysis using inductively coupled plasma-optical emission spectroscopy (ICP-OES). Multivariate optimization tools were utilized to optimize the most important parameters (pH, sorbent mass, sonication time, elution time, and eluent concentration). In addition, the method was validated using a certified reference material NIST SRM 2778 with a certified mercury concentration of 38.98 µg/kg ± 1.10 µg/kg. The well-developed method was successfully applied in real crude oil, gasoline, diesel oil, and kerosene.

## Methods

### Reagents, materials and solutions

All reagents used were of high purity and were utilized without any further processing. The ultra-pure water with a resistivity of 18 MΩ cm from a Milli-Q system (Millipore, Bedford, MA, USA) was utilized for all the preparations of calibration standards, sample solutions, and rinsing of glassware. The suprapure 70% HNO_3_, iron (II) chloride tetrahydrate (FeCl_2_.4H_2_O), iron (III) chloride hexahydrate (FeCl_3_.6H_2_O), graphite, thiourea, tetra chloroauric (III) acid (HAuCl_4_), sodium hydroxide (NaOH), potassium permanganate (KMnO_4_), hydrochloric acid, hydrogen peroxide, sulfuric acid, phosphoric acid, neodymium-iron-boron alloy magnet and ammonia were purchased from Sigma-Aldrich, South Africa. The proposed UA-m-DSPME was validated using a certified reference material NIST SRM 2778 with a certified mercury concentration of 38.98 µg/kg in fuel which was purchased from Merck, South Africa. In addition, three distinct crude oil samples were purchased from a South African petrochemical company while real gasoline, kerosine, and diesel oil samples were purchased from nearby filling stations in Johannesburg area. Every fuel sample that was purchased was labelled following Table S1**.** The viscosity of crude oil samples was reduced using 99% xylene which was purchased from Sigma-Aldrich, South Africa. Nylon microfilters (0.45 μm) were obtained from Anatech Instruments in South Africa. All glassware was washed with soap, soaked for 24 h in diluted HNO_3_ (5% v/v), extensively rinsed with Milli-Q water, and dried in an oven (Digital Scientific series 2000 oven, Scientific Engineering (Pty) Ltd, South Africa) overnight.at 100 ℃. Mercury standard (1000 mg/L) was purchased from Merck, South Africa, and was diluted to 100 mg/L to prepare external calibration standards.

### Instrumentation

The scanning electron microscopy analysis was performed on a JOEL JSM-IT 100 IntouchScope, while EDS measurements were carried out on a JOEL-made dispersive X-ray spectrometer. Transmission electron microscopy analysis was performed using JOEL JEM-1400 Plus. X-ray diffraction measurements were done using an X-ray PANalytical Pro diffractometer in the diffraction angle (2θ) window of 5⁰ to 90 ⁰ using Cu Kα irradiation. Infra-red spectra were recorded on a Brucker Optics FT-IR-4000 spectrometer. Thermogravimetric analysis was performed using a Perkinelmer Q500 thermal analyzer. Brunauer-Emmett-Teller surface area measurements were carried out at 77 K using a Quadrasorb SI automated surface area and pore size analyzer. Magnetic properties were performed using vibration sample magnetometer (VSM-Meghnatis Daghighe Kavir (MDK). Mercury concentrations were measured using Agilent Technologies 700 series ICP-OES with an axial torch orientation and the operating conditions are reported in Table S2. For sample uptake, an Agilent Technologies SPS 3 autosampler was also utilized.

### Synthesis of nanoparticles

#### Preparation of graphene oxide (GO)

Graphene oxide was prepared using Hummer’s method with some minor modifications^14^ Briefly, 50 g of NaCl was ground for 10 min with 4 g of natural graphite powder. About 3.0 g of graphite powder was added slowly into the mixture of sulphuric acid and phosphoric acid (9:1) in an ice bath while stirring continuously. Potassium permanganate (18.0 g) was then added slowly into the above mixture for 20 min. The mixture was then maintained at 50 °C in an oil bath for 24 h until its colour changed to chocolate milk. Afterwards, 400 mL of cold distilled and 3.0 mL of hydrogen peroxide was added gradually until visible bubbles were generated. The final mixture was centrifuged for 1 h to remove the supernatant and the brown precipitate was washed with deionized water and 5% hydrochloric acid. The brown precipitate was subjected to dialysis to remove excess traces of salts and acids. The final suspensions were later dried in an oven for 24 h.

#### Preparation of magnetic graphene oxide (Fe_3_O_4_/GO) nanocomposite

The carboxylate anion was dissolved by first sonicating 1 g of GO for an hour in 250 mL deionized. The reaction mixture was then placed on a heater that was surrounded by nitrogen. About 2 mmol of FeCl_3_.6H_2_O and 1 mmol of FeCl_2_.4H_2_O (dissolved in 25 mL of deionized water) were added dropwise to the graphene oxide solution after the initial addition of 5 mL of ammonia. The reaction was stirred for 5 h at 80 °C, after which the resultant product was centrifuged, cleaned with deionized water, and dried for the night at 60 °C^20^.

#### Preparation of magnetic graphene oxide coated with gold (Fe_3_O_4_/GO-Au) nanocomposite

Initially, 50 mg of Fe_3_O_4_/GO powder were sonicated for an hour at room temperature in 20 mL of deionized water. The dispersion was separated using an external magnet and then re-dispersed in 10 mL of deionized water. The final solution was made by mixing 2 mL of sodium citrate solution (0.5% w/v), 120 mL of NaOH solution (2 M), and 3 mL of HAuCl_4_ solution (20 mM). The redispersed sample from the previous stage was then added to the mixture^21^. After being mixed for an 1 h at room temperature, the finished product, Fe_3_O_4_/GO-Au) was separated using an external magnet, rinsed several times with deionized water, and dried at 60 °C.

### Ultrasound-assisted magnetic dispersive solid-phase microextraction (UA-m-DSPME) procedure

The ultrasonic-assisted magnetic dispersive solid-phase microextraction (UA-m-DSPME) method was executed by the method published by N. Raphael Biata et al.^22^with some minor modifications. At first, xylene was added to the crude oil sample to reduce its viscosity. About 5 mL of crude oil was spiked using 10 µg/L of mercury standard, and the pH of the sample solution was adjusted to 6.5 with a buffer solution. Then it was put into a glass vial with 30 mg of Fe_3_O_4_/GO-Au adsorbent. The analyte was preconcentrated for 20 min at room temperature (25 °C) in an ultrasonic bath. All experiments were carried out in triplicates. An alloy magnet made of neodymium, iron, and boron was then used to separate the adsorbent from the sample. The sample solution was decanted while the sorbent containing the target analyte was then left behind. The analyte was eluted from the sorbent using a mixture of 0.2 mol/L thiourea and 1.75 mol/L HCl. Finally, the eluted solution was separated, and mercury concentration was measured using ICP-OES.

### Optimization of UA-D-m-SPME procedure

The optimization process of the UA-m-DSPME was carried out using multivariate optimization tools. The latter approach was utilized to minimize laboratory experiments and enable the assessment of interactions between the factors^23^. The Min Table 2018 program was used for this. The most important parameters were screened using a two-level half-fractional design (2^5−1^). Eluent concentration, elution time, sorbent mass, pH, and sonication time were the most important extraction parameters that were optimized. About 17 experiments in all were produced, with the factors and levels (minimum, central, and maximum) of the experimental designs used for the optimization process shown in Table S3. Response surface methodology (RSM) was utilized to optimize the significant parameters at a 95% confidence level. The literature reports on a few RSMs, including the central composite design, the three-level factorial design, the BBD, and the Doehlert matrix. This study employed BBD to optimize the most significant variables further. These factors were assigned three levels (minimum, central, and maximum), using literature reports as our guide. These levels are shown in **Table S4**, and 27 experiments were generated. From these experiments results, we could deduce optimum conditions for each variable.

## Results and discussion

### Characterisation methods for the prepared nanoparticles

#### Fourier transform Infra-red spectroscopy (FT-IR)

**Fig. 1:**
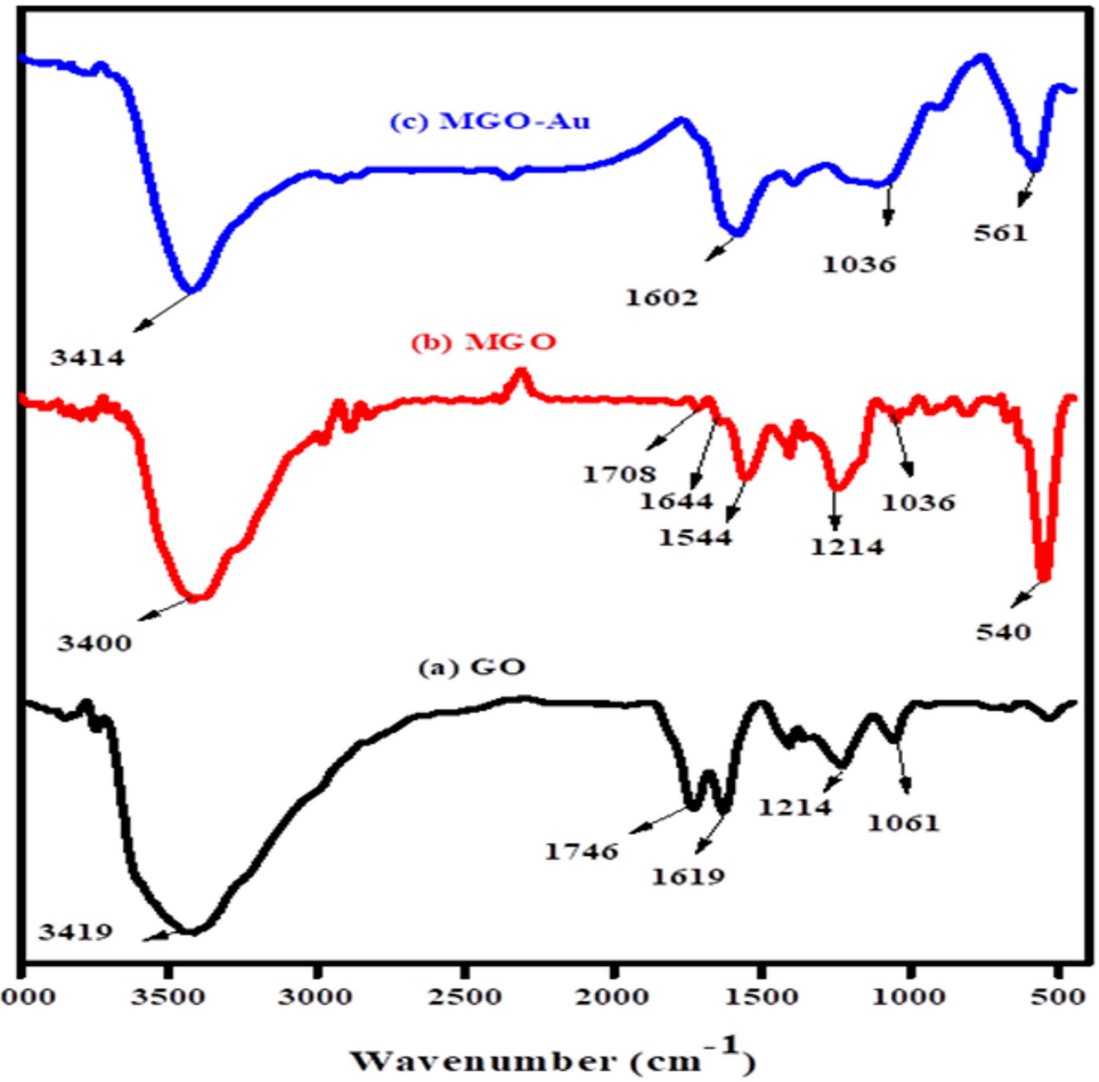
FT-IR spectra for (a) GO, (b) Fe_3_O_4_/GO, and (c) Fe_3_O_4_/GO -Au.

The composition of GO, Fe_3_O_4_/GO, and Fe_3_O_4_/GO were characterized by FT-IR. Figure 1 shows the FT-IR spectra of GO (a), Fe_3_O_4_/GO (b), and Fe_3_O_4_/GO-Au (c). In Fig. 1a a stretching vibrations peak corresponding to O-H was observed at 3419 cm^− 1^. The stretching vibrations observed at 1746 cm^− 1^ and 1619 cm^− 1^ can be assigned to C = O and C = C groups, respectively. The vibrations of alkoxy C–O stretching and O–H bending was observed at 1061 cm^− 1^ and 665 cm^− 1^, respectively^24^. This validates that GO was successfully synthesized. In Fig. 1b the asymmetric stretching vibration of C = O and the aromatic C = C vibrations of graphene give rise to a minor intensity peak at 1644 cm^− 1^ and a band at 1544 cm^− 1^, respectively. The broadband at 3400 cm^− 1^ was attributed to the O-H group, and a sharp peak at 540 cm^− 1^ was related to the stretching vibrations caused by the interactions of Fe–O–Fe in Fe_3_O_4_ NPs. After Au NPs were deposited, the Fe_3_O_4_/GO-Au core-shell nanocomposite showed less intense peaks at 582 cm^− 1^, and the band at 1544 cm^− 1^ became stronger^25^ as shown in Fig. 1c. These outcomes validate that the nanocomposite was successfully synthesized.

#### Powder-X-ray diffraction (P-XRD)

**Fig. 2:**
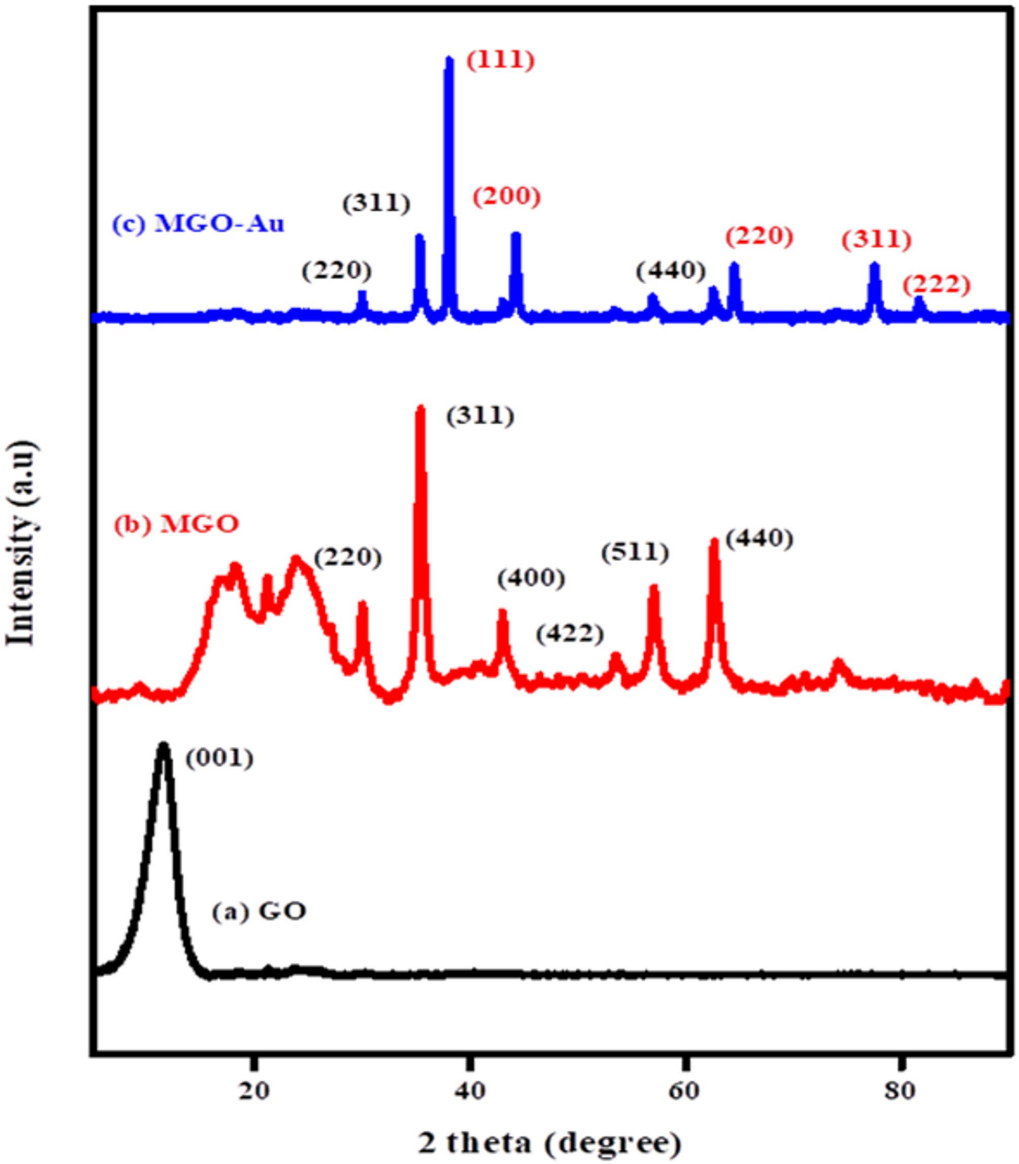
Powder X-ray diffraction patterns for (a) GO, (b) Fe_3_O_4_/GO, and (c) Fe_3_O_4_/GO-Au.

The crystalline structures and crystalline sizes of the nanocomposite were examined using P-XRD. Figure 2 (a-c) shows the diffraction patterns of GO (a), Fe_3_O_4_/GO (b), and Fe_3_O_4_/GO-Au (c). In Fig. 2 (a) the characteristic diffraction peak of GO is located at 2θ = 12.7⁰ (001). The peak was ascribed to the amorphous nature of GO and its oxygenated functional groups. In Fig. 2b characteristic diffraction peaks located at 2θ values of 30.4⁰, 35.6⁰, 43.2⁰, 53.8⁰, 57.7⁰, and 62.9⁰, respectively were observed. These were a result of the Fe_3_O_4_ cubic lattice (220), (311), (400), (422), (511), and (440) crystal planes. It is worth noting that the peak corresponding to GO vanished due to the increased nanoparticle loadings that damage of the original lattice structure of the GO substrate and depression of the carbon signal^26^. Gold nanoparticles (Fig. 2c) are visible at 2θ:38.0⁰, 44.2⁰, 64.5⁰, 77.5⁰, and 81.5⁰, which correspond to the (111), (200), (220), (311), and (222) planes, in that order^27^. These results verify that the Fe_3_O_4_/GO-Au nanocomposite was appropriately synthesized. Using Sherrer equation, the average crystallite size of GO, Fe_3_O_4_-GO and Fe_3_O_4_-GO-Au were determined to be 13.34 nm.

#### Scanning electron microscopy-energy dispersive spectroscopy (SEM-EDS)

**Fig. 3:**
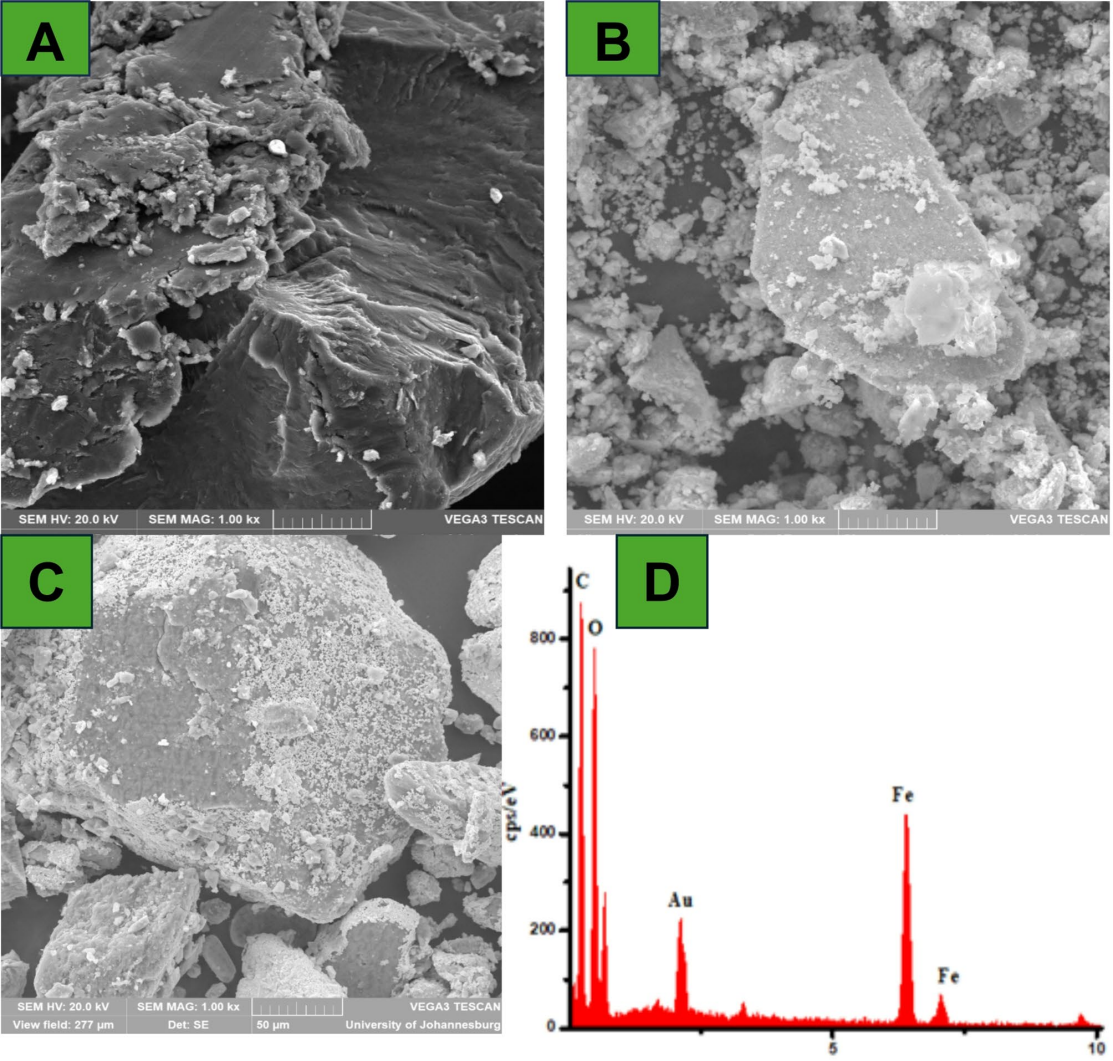
Scanning electron microscopy for (a) GO, (b) Fe_3_O_4_/GO, (c) Fe_3_O_4_/GO-Au. And (d) EDS for Fe_3_O_4_/GO-Au.

A scanning electron microscope (SEM) in conjunction with energy dispersive spectroscopy (EDS) was used to further examine the morphology and elemental composition of the synthesized materials (GO, Fe_3_O_4_/GO, and Fe_3_O_4_/GO-Au core-shell nanocomposite), as shown in Fig. 3 (A-C). According to the results, GO sheets were formed, as shown in Fig. 3A. Adding Fe_3_O_4_ nanoparticles to GO caused it to appear as brilliant spots evenly distributed across its surface, in contrast to the smooth surface of GO (Fig. 3B). The Fe_3_O_4_/GO-Au formation is shown in Fig. 3C. On the surface of the GO nanosheet, the Au and Fe_3_O_4_ nanoparticles are exhibited as spherical formations with an average size of 14 nm. Furthermore, EDS (Fig. 3D) confirmed the successful synthesis of Fe_3_O_4_/GO-Au due to the presence of Fe, C, O, and Au. This also confirms the successful synthesis of the Fe_3_O_4_/GO-Au nanocomposite.

#### Transmission electron microscopy (TEM)

Figure S1(A-D) presents TEM results of the synthesized nanocomposite. The thin, layered structure of graphene oxide sheets is characterized by folded wrinkles, and their vast surface area makes them particularly advantageous for loading nanoparticles (NPs) (Fig. S1A). The Fe_3_O_4_ NPs anchored on GO sheets created a huge number of Fe_3_O_4_ NPs with various sizes, as seen in Fig. S1B. By adding Fe_3_O_4_ NPs to the GO sheets, a stacked graphitic structure cannot form, and the composites have magnetic characteristics that make them easily recyclable. The typical shape of the synthesized nanocomposite is depicted in the TEM image (Fig. S1C). The produced nanoparticles are visible as they are distributed across the GO surface, displaying lighter iron oxide particles and darker gold nanoparticles. The electron density of gold nanoparticles gives them a deeper color^21^. Image J software was used to calculate the particle size distribution of the synthesized Fe_3_O_4_/GO-Au, as shown in Fig. S1D. Corresponding to the XRD and SEM data, an average particle size of 14 nm was obtained.

Figure S1 (A-C) displays insets of GO, Fe_3_O_4_/GO, and Fe_3_O_4_/GO-Au selected area electron diffraction (SAED) patterns. Figure S1A SAED patterns insert show that GO has an amorphous pattern. Figure S1B shows that the Fe_3_O_4_/GO nanoparticles were extremely crystalline and could be accurately indexed to the cubic structure of pure Fe_3_O_4_^25^ A polycrystalline pattern for Fe_3_O_4_/GO-Au nanocomposite is also shown in the inset in Fig. S1C The SAED data correlates very well with the P-XRD patterns.

#### Brunauer-Emmett-Teller (BET)

**Fig. 4:**
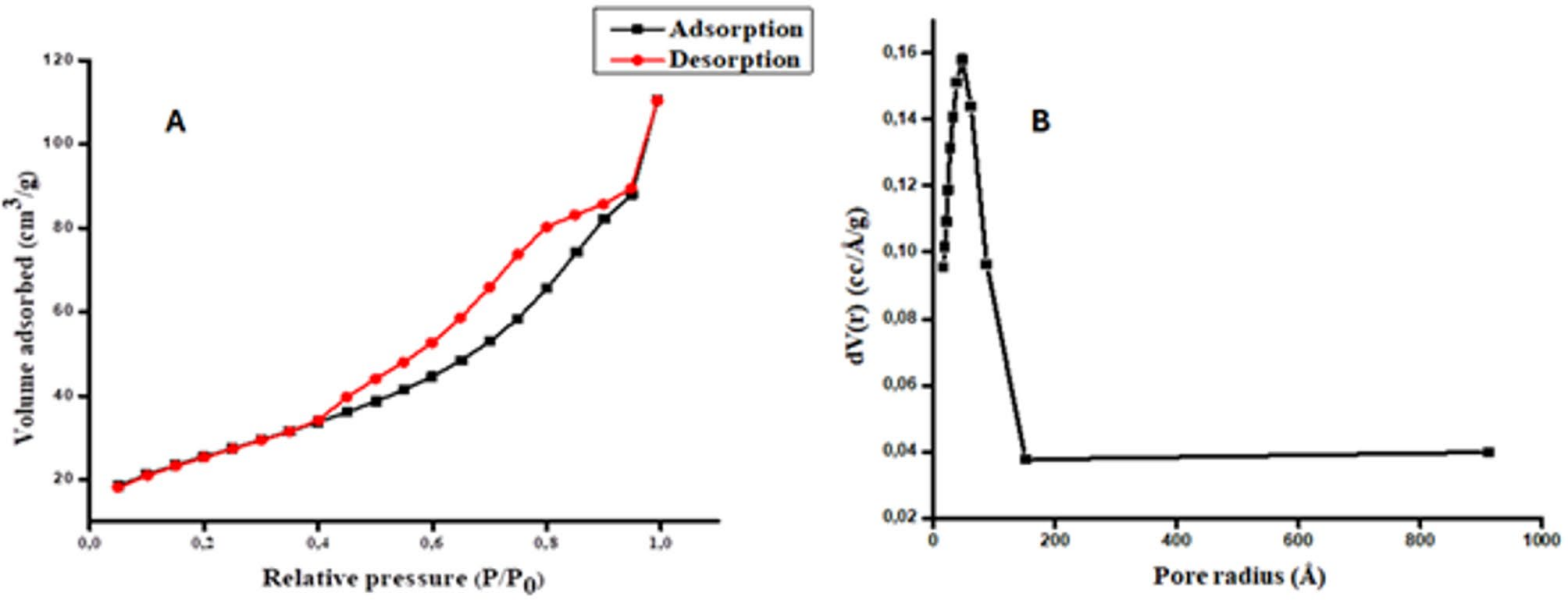
The (A) nitrogen adsorption isotherm curve and (B) pore size distribution of Fe_3_O_4_/GO-Au core shell nanocomposite.

Brunauer Emmett Teller (BET) measurements were performed to determine the specific surface area, surface functional group, and pore size distribution of the synthesized nanocomposite. The porosity was determined using the nitrogen adsorption isotherm, and the pore size distribution was determined using the Barrett-Joyner-Halenda (BJH) method (Fig. 4A & B). The surface areas of GO, Fe_3_O_4_/GO nanocomposite, and Fe_3_O_4_/GO-Au core-shell nanocomposite were 32.187, 42.222, and 57.920 m^2^ g^− 1^, respectively, according to Table S5. The surface area of GO increased when magnetic and gold nanoparticles were incorporated on its surface. The main advantages of having high surface area of adsorbent is the increased contact between the adsorption sites of Fe_3_O_4_-GO-Au and analyte thus, improving the adsorption capacity of Fe_3_O_4_-GO-Au^28^. The isotherm (Fig. 4A) displays characteristics of an H3 hysteresis loop in the range of 0.4–0.9 (P/P0), or a type IV isotherm. Moreover, Fig. 4B illustrates that the average pore size distribution of Fe_3_O_4_/GO-Au core-shell nanocomposite was at around 17–47 nm, indicating that the material was mesoporous as well^29^.

**Fig. 5:**
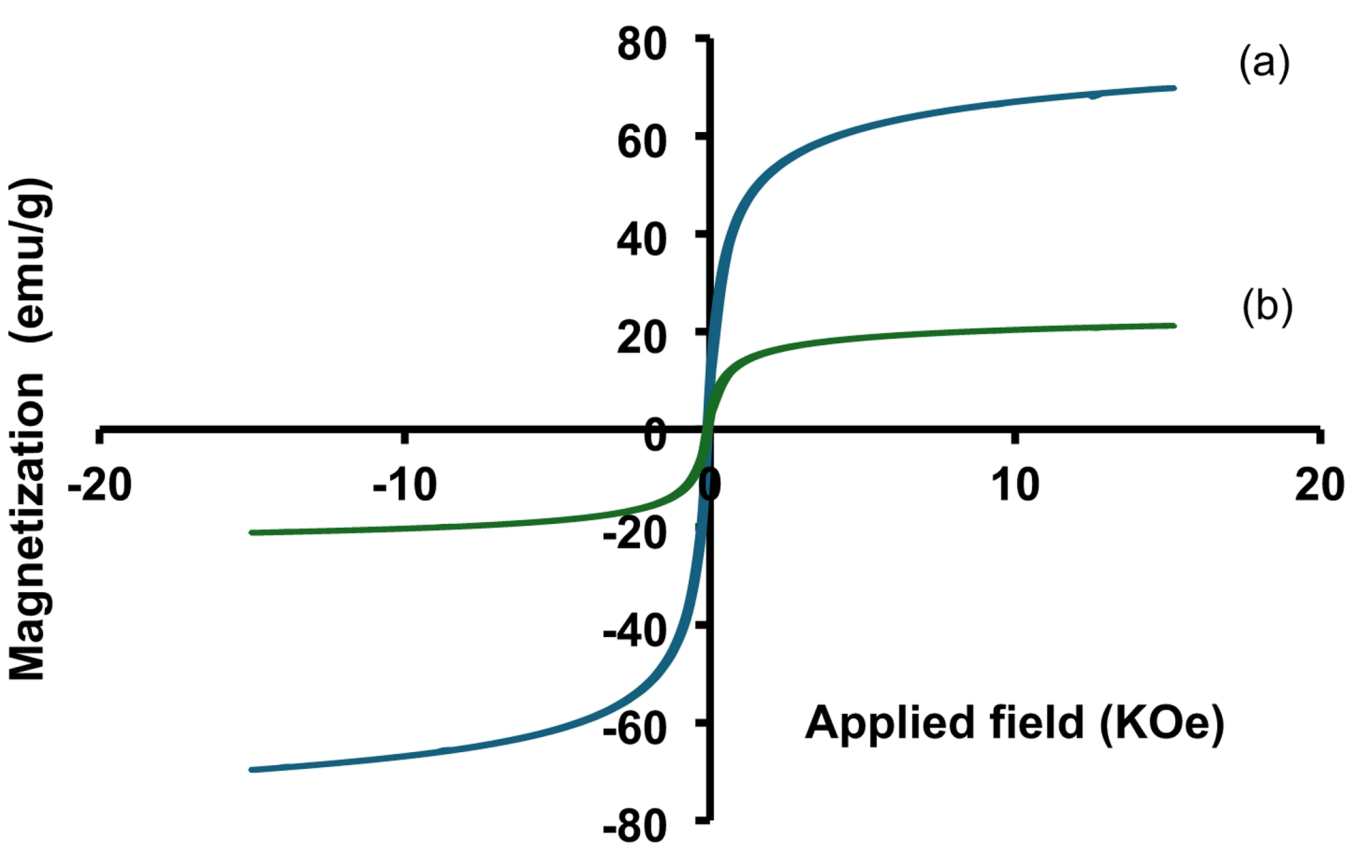
Magnetic hysteresis loops of (a) Fe_3_O_4_-GO and (b) Fe_3_O_4_-GO-Au.

The magnetic characteristic of Fe_3_O_4_-GO (a) and Fe_3_O_4_-GO-Au (b) were evaluated using vibrating sample magnetometer (VSM) at room temperature. The magnetic field was measured between − 80 and 80 Oe and the obtained hysteresis loops were S shaped (Fig. 5). For Fe_3_O_4_-GO nanocomposite, the saturation magnetization (Ms) and the coercivity of hysteresis loop (Hc) were determined as 69.8 emu/g and − 0.013 kOe, respectively. The magnetic remanence (Mr) of the nanocomposite was 5 emu/g. On the other hand, the saturation magnetization (Ms) of Fe_3_O_4_-GO-Au nanocomposite was determined to be 21 emu/g. The magnetic remanence and coercivity of the nanocomposite were determined to be 4.6 emu/g and − 0.013 kOe, respectively. The obtained saturation magnetization values and VSM curves confirm that all mentioned materials represent superparamagnetic properties and can be isolated from the solution by employing an external magnetic field. It can be noted that the saturation magnetization of Fe_3_O_4_-GO-Au was reduced due to the coating of gold on the surface of Fe_3_O_4_-GO.

### Choice of eluent

For the desorption of mercury from different nanomaterials, HCl is typically recommended as an eluent of choice in the literature studies. However, in this study it was necessary to first screen how adding various trapping agents would affect the results. This was accomplished by analyzing a NIST SRM 2778 with a certified mercury concentration of 38.98 µg/kg ± 1.10 µg/kg under these conditions: sample mass: 30 mg; pH: 6; sonication period: 30 min; elution period: 6 min. The following three eluent combinations were examined: 10% HCl, 0.05 mol/L Au + 10% HCl, and 0.05 mol/L thiourea + 10% HCl. Figure S2 displays the findings. This figure shows that increasing the thiourea concentration as a trapping agent in HCl results in higher recoveries (101%). The thiourea concentration was maintained at 0.2 mol/L while the HCl content was further optimized.

### Multivariate optimization

#### Two-level half-factorial design

**Fig. 6:**
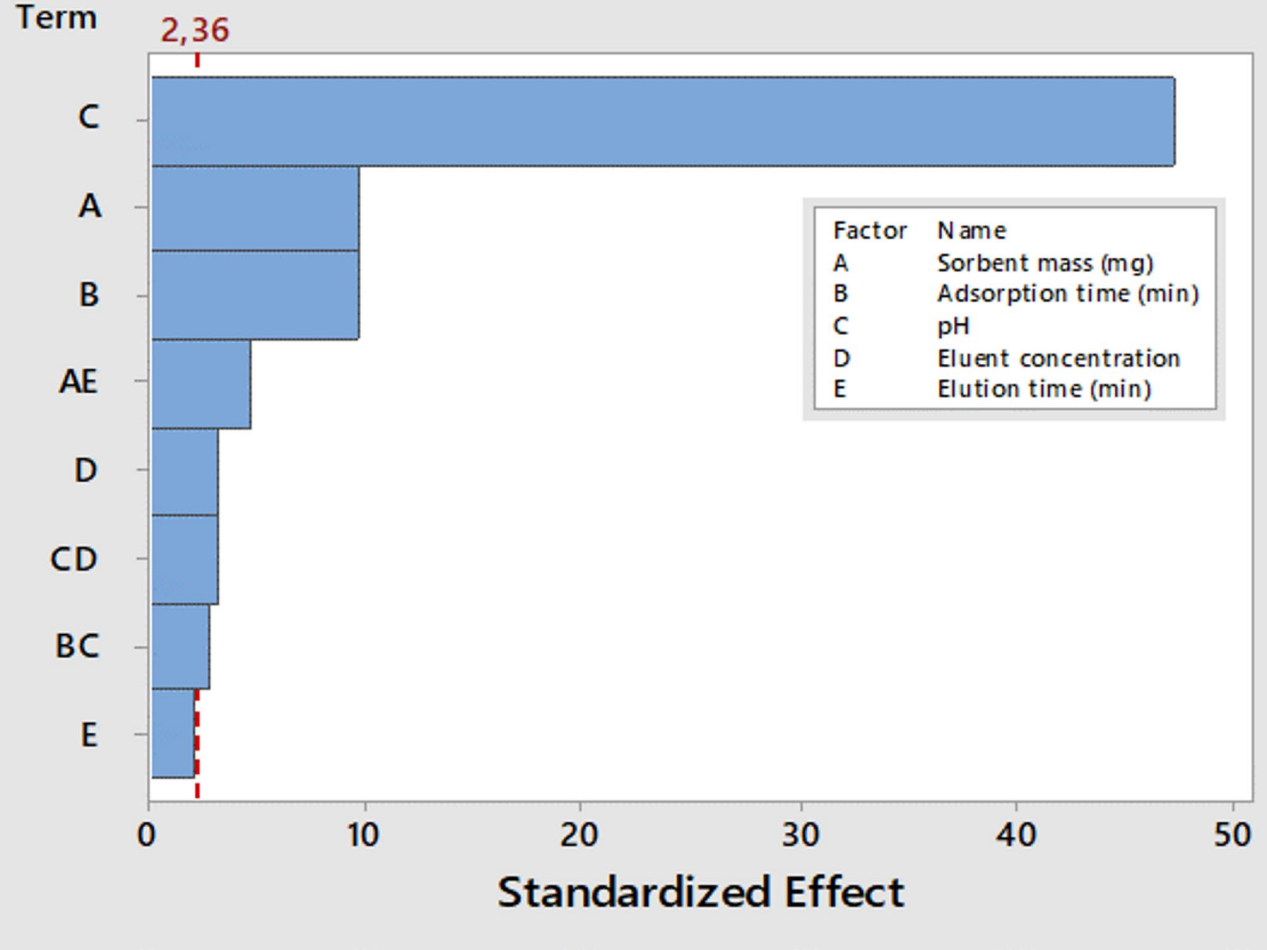
Two-level half factorial design.

The effects of several experimental parameters, including sorbent mass, pH, adsorption time, eluent concentration, and elution time, were examined using a two-level half-factorial design. Table S6 presents the parameters, number of experiments, experimental conditions, and outcomes of the two-level half-factorial design. The primary effects and their interactions were assessed using the analysis of variance (ANOVA) (Table S7), which was displayed as Pareto charts (Fig. 6). The bar length relates to the absolute value of the estimated impacts, while the vertical line shows the 95% confidence level. Comparing the proportional importance of the impact is made easier by the bar length. According to the ANOVA results, the eluent concentration, pH, adsorbent mass, and adsorption period were all significant at the 95% confidence level (bar length is greater than the reference line). The Box-Behnken design further optimized the critical factors.

#### Further optimization using response surface methodology

Response surface methodology based on the Box-Bohnken design was used to determine the optimal conditions of the relevant factors. Table S8 displays the Box-Bohnken design matrix along with the analytical response values. For all the tests, the Hg (II) concentration was maintained at 10 µg/L and the statistically insignificant factor elution duration was kept constant at 6 min. This is how the model was described:

The design model led to the coefficient of determination (R^2^ and adjusted R^2^ values of 0.9035 and 0.8191, respectively, indicating that it was appropriate for optimizing the preconcentration, according to the ANOVA data (Table S9). It is not significant to pure error, as indicated by the F-value of 35.8 for the lack of fit.

**Fig. 7:**
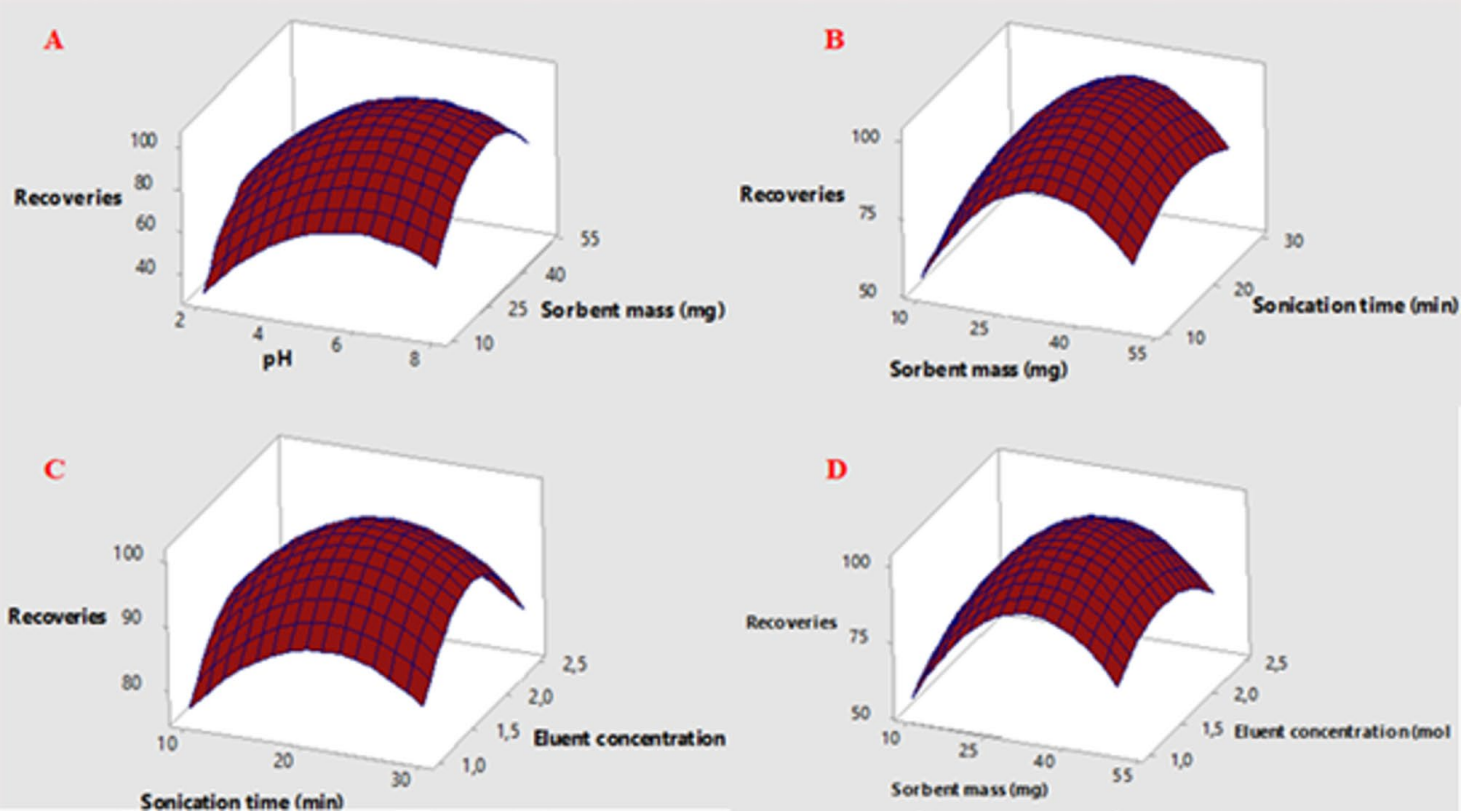
Surface responses total Hg versus pH. Sorbent mass, sorbent mass. Sonication time, sonication time. Eluent concentration and Sorbent mass. Eluent concentration.

Figure 7 (A-D) illustrates how the two factors interact to provide the highest adsorption capacity. The analytical response rises as pH rises, but it falls at higher pH levels (pH > 7), as seen in Fig. 7A. Mercury creates a variety of species at pH values higher than 7, including stable mercuric hydroxide, which prevents the production of Au-Hg complexes, which explains this phenomenon^30^. The surface plots, as presented in Fig. 7B & D, demonstrate that the analytical response both rises and falls with increasing sorbent mass. One could argue that insufficient sorbent causes an incomplete extraction. However, high concentrations cause the sorbent to become saturated, necessitating a considerable volume of eluent to fully desorb the analyte from the adsorbent’s surface^31^. The duration of sonication was examined within the range of 10 to 30 min. Shorter sonication intervals result in lower recoveries because of inadequate adsorption, as Fig. 7B illustrates, but longer times do not boost stability.

Response surface plots and the quadratic equation showed that sample pH of 6.5, sorbent mass of 30 mg, sonication time of 20 min, and eluent concentration of 1.75 mol/L are the values that yield the best response.

### Greenness assessment of the newly developed m-SPME procedure

Analytical chemistry relies heavily on this assessment section since it assesses the environmental impact of every established process. For these reasons, several metric instruments with differing degrees of comprehensiveness have been created. They are predicated on the inclusion of additional criteria, and the generic response of the assessment varies greatly in terms of both complexity and appearance^32^. When evaluating the greenness of any analytical procedure, the analytical greenness approach (AGREEprep) has received a lot of attention. Ten factors make up this statistic, which is used to evaluate the environmental impact of sample preparation. Every criterion has a default weight of its own (Table S10). A pictogram representing the results is displayed (Fig. S3).

The pictogram (Fig. S3) displays a total score of 0.64, exceeding the minimum requirement of 0.6 for a method to be classified as environmentally friendly^14^. This implies that the recently created m-SPME is more environmentally friendly. For the analytical approach to get the desired result of 1, a few steps still need to be improved. There is room for improvement in stages 1, 4, 5, 7, and 9. Because the extraction was done in a lab setting, criteria 1 received a low score. Criteria 4 did not meet expectations due to the high waste output of the method. Moreover, the sample size of 5 mL was a little excessive, making Criterion 5 subpar. ICP-OES, an energy-intensive post-sample preparation approach, was used instead of the recently developed method, which did not encourage automation. Consequently, criteria 7 and 9 were likewise poor. Therefore, this recently established technology can be a lot greener if the inadequate criteria can be modified.

### Validation of the proposed method

The accuracy of the proposed method was assessed using NIST SRM 2778 under optimal conditions. Figure S4 compares the precision of the three nanomaterials: GO, Fe_3_O_4_/GO, and Fe_3_O_4_/GO-Au. Percentage recoveries were obtained to be 35%, 40%, and 105%, respectively. Additionally, the sensitivity, limit of detection, limit of quantification, and precision of the proposed method were further assessed to investigate its analytical performance. The calibration curve was obtained by analyzing seven standard solutions (0–100 µg/L). A correlation coefficient (R^2^ of 0.9996 indicated the presence of linearity between 0.1 and 100 µg/L. The preconcentration factor, which is the ratio of the calibration curve before and after preconcentration was 255 for the UA-m-DSPME. The limits of detection and quantification were 0.035 µg/L and 0.119 µg/L, respectively. These limits were specified as 3SD/m and 10 SD/m, where SD is the standard deviation of ten replicate measurements of blank samples and m is the calibration slope. The precision of the suggested method was investigated by analyzing a series of standard solutions containing 50 µg/L of mercury ions. The repeatability and interday precision of the method.

were 3.5%, and 4.0%, respectively for the determination of 50 µg/L of mercury ions as relative standard deviation (RSD%).

### Comparison of the developed method with other literature reports

The analytical performance of the newly developed method was compared with other literature findings (Table 1). The analytical features of the newly developed procedure were comparable with other literature findings. A good accuracy of 105% was demonstrated by the recently developed analytical approach which is within the permissible analytical range and what most methods reported. Additionally, the method demonstrated good precision, albeit showing LODs. This can be justified using ICP-OES, which is widely known for having low sensitivity toward the target analyte^33^. Compared to other published methods, the new method had greater preconcentration factors (PF), which made it a favourable substitute for the preconcentration of total mercury. Even though the method demonstrated a long extraction time, to our best knowledge, it is the only method that has been assessed for greenness compared to the other extraction methods for total mercury determination.

**Table 1:** Comparison of the figures of merits between newly developed m-SPE with other SPE report on various matrices.

Sample preparation	Adsorbent	Adsorbent mass (mg)	Contact time (min)	Sample	Analytical technique	LOD (µg/L)	LDR (µg/L)	PF	Greenness assessment	Accuracy (%)	Reusability	RSD (%)	REF
m-SPME	MGO-Au	30	20	Fuel oils	ICP-OES	0.0350	0.1–100	255	0.64	105	7	4.0	This work
m-SPE	DT–Fe_3_O_4_	80	5	Environmental	CV-AAS	0.0500	2–70	250	N/R	94.2	N/R	10.1	^34^
S-D-IL-µSPE	NG-COOH	10	3	Blood	FI-CV-AAS	0.0100	N/R	22	N/R	97–103	N/R	4.0	^35^
m-SPE	Fe_3_O_4_ @UiO-66-SH	20	15	Water and fish	ICP-MS	0.0026	20-1000	45.7	N/R	84.5–96.8	10	5.7	^36^
m-SPE	Fe_3_O_4_@SiO_2_@AMPTs	10	15	Water	DMA	0.0017	N/R	100	N/R	94.9–107	N/R	2.4	^37^
m-SPE	DPTH@MGO	4	3	Water	ICP-OES	0.0500	0.2–1000	3	N/R	93–105	N/R	1.6	^38^
DSPME	MIP-PEHA	50	1	Food	HPLC-MS	N/R	N/R	N/R	0.62	N/R	N/R	N/R	^39^
SPE	Octadecyl C18	N/R	180	Textile wastewater	HPLC/MS	2.6–30	N/R	N/R	0.61	81.6-103.2	N/R	< 10	^40^
m-SPME	GO-MNPs	10	15	AY 23 dye	UV-vis	0,0026	0.2-5	N/R	0,50	99,7-100.4	N/R	0.5–2.9	^41^

### Interference studies

To examine the selectivity of the suggested UA-m-DSPME approach, standard solutions containing 10 µg/L Hg (II) and other metal cations that are typically found in fuel oils (200 µg/L) were prepared and analyzed using the established method. Among the metallic cations are Ni (II), Fe (II), Cd (II), Pb (II), Co (II), Cu (II), and Mn (II). The highest concentration of interfering ions that results in a recovery of less than 95% of Hg (II) was designated as the tolerance limit in this investigation. The recoveries varied from 95 to 99% for Fe_3_O_4_/GO-Au (Table S11), indicating that the selectivity of the method is comparatively good. The same process was also used to examine the selectivity of GO and Fe_3_O_4_/GO. Nevertheless, recoveries obtained using the two materials were below 95% indicating that their selectivity was poor.

### Reusability studies

When considering industrial applications, the adsorbent’s reusability is essential. Fig. S5 displays the reusability investigations of Fe_3_O_4_/GO-Au. According to the figure, the adsorbent can be used up to seven times, with a 93–100% mercury recovery rate. As a result, it can be said that the adsorbent was reasonably reusable for a long time when it came to mercury. The magnetic characteristic of the reused Fe_3_O_4_-GO-Au nanocomposite was confirmed using vibrating sample magnetometer (VSM). The reused nanocomposite still showed a magnetic hysteresis loop which was S shaped (Fig. S7). This confirms that the magnetic nanocomposite still retained its magnetic properties after being reused for a long time. The saturation magnetization (Ms) of the reused nanocomposite was still at around 21 emu/g, which indicates that the nanocomposite was still magnetic and can be separated using an external magnet. In 2014, Jing Hu and coworkers^32^ recorded an equivalent quantity of cycles with the same material but their focus was on the reduction of 4-nitrophenol.

### Application of UA-m-DSPME procedure in real samples

Real crude oil, gasoline, diesel oil, and kerosine samples were subjected to the recently developed ultrasonic-assisted magnetic dispersive solid phase microextraction (UA-m-DSPME). Table S1 was followed in labelling the actual fuel samples. Table 2 displays the obtained total concentrations of mercury. Regarding the mercury contamination of the fuel samples, the table shows a pattern. Samples of crude oil had the highest level of contamination, followed by those of diesel, gasoline, and kerosene. This is consistent with some literature reports. This indicates that the recently developed preconcentration process is reliable and a good substitute for existing techniques.

When compared to fuel oils from other nations, South African fuel oils don’t have significant mercury contamination. In diesel oil, Wu and coworkers’ investigation yielded a total mercury content of 0.83 µg/g^42^. Uddin and coworkers repeated an intriguing study about the extraction of total mercury from crude oil. They found that Arabian crude oil had a total mercury concentration of 0.18 µg/kg using their method^43^.This demonstrates that South African fuel oils contain mercury at levels comparable to those of other nations.

**Table 2:** Total mercury concentration levels (µg/g) in real crude oil, gasoline, diesel oil, and kerosene.

Sample type	Concentration (µg/g)
COS 1	0.410 ± 0.03
COS 2	0.560 ± 0.09
COS 3	0.490 ± 0.15
GS 1	0.310 ± 0.07
GS 2	0.425 ± 0.08
GS 3	0.480 ± 0.01
DS 1	0.500 ± 0.05
DS 2	0.431 ± 0.04
DS 3	0.550 ± 0.01
KS 1	0.089 ± 0.30
KS 2	0.090 ± 0.50
KS 3	0.081 ± 0.004

## Conclusion

In conclusion, the magnetic graphene oxide coated with gold nanocomposite (Fe_3_O_4_/GO-Au) was successfully prepared, characterized, and applied as nano adsorbent for selective extraction and preconcentration of trace mercury in crude oil, diesel, gasoline, and kerosene samples before ICP-OES analysis. The newly developed method showed good accuracy, intraday, and preconcentration factor of 105%, 3.5% and 255, respectively. Compared to other extraction method proved to be environmentally friendly with an AGREEprep score of 0.64. Furthermore, Fe_3_O_4_/GO-Au enhanced magnetic-assisted separation of the adsorbent, and high selectivity towards mercury. Because of the relatively high PF, trace amounts of mercury ions at µg/g levels in high-volume samples can be determined by the Fe_3_O_4_/GO-Au. For the preconcentration of total mercury in fuel oils, the synthesized adsorbent may be reused seven times.

Currently, there is no legislation concerning the quantity of mercury permitted in fuel samples, but South African fuels contained mercury concentrations which were like the rest of the other countries. Therefore, this study offered a new powerful way to design other efficient nano adsorbent (Fe_3_O_4_/GO-Au) to monitor and determine trace amounts of mercury in fuel oils.

## Electronic supplementary material

Below is the link to the electronic supplementary material.

Supplementary Material 1

## Data Availability

All data generated or analysed during this study are included in this published article and its supplementary information file.
