# Supplementary material for: Ultrasound assisted magnetic dispersive solid phase microextraction for Hg determination in fuel oils using inductively coupled plasma optical emission spectroscopy
Source: Sci Rep. 2025 May 13;15:16557. doi: 10.1038/s41598-025-01447-8 (PMC12075474; doi:10.1038/s41598-025-01447-8)
Supplement: Supplementary file 1 — Supplementary Material 1 [file 41598_2025_1447_MOESM1_ESM.docx]

**Ultrasound assisted magnetic dispersive solid phase microextraction for Hg determination in fuel oils using inductively coupled plasma optical emission spectroscopy**

Mxolisi J Kiwanuka^a^, Philiswa N Nomngongo^b^, Nomvano Mketo^a^

*^a^Department of Chemistry, College of Science, Engineering and Technology (CSET), University of South Africa, Florida Science Campus, 1709, Johannesburg, South Africa*

*^b^Department of Chemical Sciences, Faculty of Science, University of Johannesburg, Johannesburg, South Africa*

*Corresponding author: Nomvano Mketo (Email address: nomvano.mketo@gmail.com or mketon@unisa.ac.za; Tel: +27114712032)*

**Supplementary**

**Table S1:** Different types of fuel oils and their abbreviations.

| **Sample type** | **Sample 1** | **Sample 2** | **Sample 3** |
| --- | --- | --- | --- |
| Crude oil | COS 1 | COS 2 | COS 3 |
| Gasoline | GS 1 | GS 2 | GS 3 |
| Diesel oil | DS 1 | DS 2 | DS 3 |
| Kerosine | KS 1 | KS 2 | KS 3 |

**Table S2:** Operating parameters of ICP-OES for mercury analysis.

| **Agilent ICP-OES parameters** | **Instrumental conditions** |
| --- | --- |
| RF power (W) | 1200 |
| Auxiliary gas Flow (L/min) | 1.5 |
| Plasma gas (Arg) flow (L/min) | 15 |
| Pump speed (rpm) | 85 |
| Peri-pump speed analysis (rpm) | 15 |
| Sample uptake delay (s) | 15 |
| Stabilisation time (s) | 15 |
| Nebulizer (L/min) | 0.75 |
| Elemental wavelengths (nm) | Hg (184.887) |

**Table S3:** Two-level half factorial (2^5-1^) experimental design.

| **Factor** | **Minimum (-)** | **Central (0)** | **Maximum (+)** |
| --- | --- | --- | --- |
| Sorbent mass (mg) | 10 | 30 | 50 |
| Sonication time (min) | 10 | 35 | 60 |
| pH | 3 | 6 | 9 |
| Eluent concentration (mol/L) | 0.1 | 0.55 | 1.0 |
| Elution time (min) | 3 | 4 | 5 |

**Table S4:** Response methodology based on BBD experiment design.

| **Factor** | **Minimum (-)** | **Central (0)** | **Maximum (+)** |
| --- | --- | --- | --- |
| Sorbent mass (mg) | 10 | 30 | 50 |
| pH | 2 | 5 | 8 |
| Sonication time (min) | 10 | 20 | 30 |
| Eluent concentration (mol/L) | 1.0 | 1.75 | 2.5 |

**Table S5:** The BET results of the GO, Fe_3_O_4_/GO and Fe_3_O_4_/GO-Au core shell nanocomposite.

| **Sample** | **Surface area (m^2^/g)** |
| --- | --- |
| GO | 42.222 |
| Fe_3_O_4_/GO | 32.187 |
| Fe_3_O_4_/GO-Au | 57.962 |

**Table S6:** The effect of varying sorbent mass, sample pH, sonication, eluent concentration, and elution time and centrifugation time, on the UA-m-DSPME of real crude oil sample to achieve high percentage recoveries of total Hg. Replicates (n=3).

| **Std Order** | **Run Order** | **Center Pt** | **Blocks** | **Sorbent mass (mg)** | **Sonication time (min)** | **pH** | **Eluent concentration (mol/L)** | **Elution time (min)** | **Recoveries (%)** |
| --- | --- | --- | --- | --- | --- | --- | --- | --- | --- |
| 1 | 1 | 1 | 1 | 10 | 10 | 3 | 0.1 | 5 | 38.0 |
| 2 | 2 | 1 | 1 | 50 | 10 | 3 | 0.1 | 3 | 49.0 |
| 3 | 3 | 1 | 1 | 10 | 60 | 3 | 0.1 | 3 | 48.0 |
| 4 | 4 | 1 | 1 | 50 | 60 | 3 | 0.1 | 5 | 58.0 |
| 5 | 5 | 1 | 1 | 10 | 10 | 9 | 0.1 | 5 | 87.7 |
| 6 | 6 | 1 | 1 | 50 | 10 | 9 | 0.1 | 3 | 90.0 |
| 7 | 7 | 1 | 1 | 10 | 60 | 9 | 0.1 | 3 | 89.9 |
| 8 | 8 | 1 | 1 | 50 | 60 | 9 | 0.1 | 5 | 99.9 |
| 9 | 9 | 1 | 1 | 10 | 10 | 3 | 1.0 | 5 | 50.0 |
| 10 | 10 | 1 | 1 | 50 | 10 | 3 | 1.0 | 3 | 55.0 |
| 11 | 11 | 1 | 1 | 10 | 60 | 3 | 1.0 | 3 | 49.9 |
| 12 | 12 | 1 | 1 | 50 | 60 | 3 | 1.0 | 5 | 60.0 |
| 13 | 13 | 1 | 1 | 10 | 10 | 9 | 1.0 | 5 | 79.9 |
| 14 | 14 | 1 | 1 | 50 | 10 | 9 | 1.0 | 3 | 89.0 |
| 15 | 15 | 1 | 1 | 10 | 60 | 9 | 1.0 | 3 | 95.0 |
| 16 | 16 | 1 | 1 | 50 | 60 | 9 | 1.0 | 5 | 104.0 |
| 17 | 17 | 0 | 1 | 10 | 35 | 6 | 0.55 | 4 | 89.0 |

**Table S7:** Analysis of variance of half factorial design for UA-m-DSPME.

| **Source** | **DF** | **Adj. SS** | **Adj. MS** | **F-value** | **P-value** |
| --- | --- | --- | --- | --- | --- |
| Model | 7 | 7175.4 | 1025.1 | 5.79 | 0.009 |
| Linear | 5 | 5354.3 | 1070.9 | 6.05 | 0.10 |
| Sorbent mass | 1 | 175.6 | 175.1 | 0.99 | 0.345 |
| Ultrasonication time (min) | 1 | 2002.6 | 2002.6 | 11.32 | 0.008 |
| pH | 1 | 1580.1 | 1580.1 | 8.93 | 0.015 |
| Eluent concentration (mol/L) | 1 | 798.1 | 798.1 | 4.51 | 0.063 |
| Elution time (min) | 1 | 798.1 | 798.1 | 4.51 | 0.063 |
| 2-way interactions | 2 | 1821.1 | 910.6 | 5.15 | 0.032 |
| Sorbent mass (mg)*Eluent concentration (mol/L) | 1 | 1105.6 | 1105.6 | 6.25 | 0.034 |
| Sonication time (min)*Elution time (min) | 1 | 715.6 | 715.6 | 4.04 | 0.075 |
| Error | 9 | 1592.7 | 177.0 |  |  |
| Curvature | 1 | 220.7 | 220.7 | 1.29 | 0.289 |
| Lack of fit | 8 | 1372.0 | 171.5 |  |  |
| Total | 16 | 8768.1 |  |  |  |

**Table S8:** The effect of varying pH, sonication time, sorbent mass, and eluent concentration, while keeping elution time constant in further optimization using BBD. Experimental conditions: 5 minutes elution time (n =3).

| **Std Order** | **Run Order** | **Pt Type** | **Blocks** | **Sorbent mass (mg)** | **pH** | **Sonication time (min)** | **Eluent concentration (mol/L)** | **Recoveries (%)** |
| --- | --- | --- | --- | --- | --- | --- | --- | --- |
| 1 | 1 | 2 | 1 | 10 | 2 | 20 | 1.75 | 33.00 |
| 2 | 2 | 2 | 1 | 50 | 2 | 20 | 1.75 | 56.00 |
| 3 | 3 | 2 | 1 | 10 | 8 | 20 | 1.75 | 60.00 |
| 4 | 4 | 2 | 1 | 50 | 8 | 10 | 1.75 | 67.00 |
| 5 | 5 | 2 | 1 | 30 | 5 | 30 | 1.00 | 78.90 |
| 6 | 6 | 2 | 1 | 30 | 5 | 10 | 1.00 | 81.00 |
| 7 | 7 | 2 | 1 | 30 | 5 | 30 | 2.50 | 79.00 |
| 8 | 8 | 2 | 1 | 30 | 5 | 20 | 2.50 | 85.00 |
| 9 | 9 | 2 | 1 | 10 | 5 | 20 | 1.00 | 53.00 |
| 10 | 10 | 2 | 1 | 50 | 5 | 20 | 1.00 | 67.80 |
| 11 | 11 | 2 | 1 | 10 | 5 | 20 | 2.50 | 52.10 |
| 12 | 12 | 2 | 1 | 50 | 5 | 10 | 2.50 | 69.90 |
| 13 | 13 | 2 | 1 | 30 | 2 | 10 | 1.75 | 54.00 |
| 14 | 14 | 2 | 1 | 30 | 8 | 30 | 1.75 | 80.00 |
| 15 | 15 | 2 | 1 | 30 | 2 | 30 | 1.75 | 53.00 |
| 16 | 16 | 2 | 1 | 10 | 8 | 10 | 1.75 | 84.00 |
| 17 | 17 | 2 | 1 | 50 | 5 | 10 | 1.75 | 48.00 |
| 18 | 18 | 2 | 1 | 10 | 5 | 30 | 1.75 | 70.00 |
| 19 | 19 | 2 | 1 | 50 | 5 | 30 | 1.75 | 69.00 |
| 20 | 20 | 2 | 1 | 30 | 5 | 30 | 1.75 | 75.00 |
| 21 | 21 | 2 | 1 | 30 | 2 | 20 | 1.00 | 51.00 |
| 22 | 22 | 2 | 1 | 30 | 8 | 20 | 1.00 | 87.00 |
| 23 | 23 | 2 | 1 | 30 | 2 | 20 | 2.50 | 40.00 |
| 24 | 24 | 2 | 1 | 30 | 8 | 20 | 2.50 | 89.00 |
| 25 | 25 | 0 | 1 | 30 | 5 | 20 | 1.75 | 99.90 |
| 26 | 26 | 0 | 1 | 30 | 5 | 20 | 1.75 | 100.08 |
| 27 | 27 | 0 | 1 | 30 | 5 | 20 | 1.75 | 101.00 |

**Table S9:** Analysis of variance of BBD for UA-m-DSPME.

| **Source** | **DF** | **Adj. SS** | **Adj. MS** | **F-value** | **P-value** |
| --- | --- | --- | --- | --- | --- |
| Model | 14 | 9051.7 | 646.55 | 10.70 | 0.001 |
| Linear | 4 | 5758.8 | 1439.70 | 23.84 | 0.015 |
| Sonication time (min) | 1 | 928.0 | 927.95 | 15.36 | 0.004 |
| pH | 1 | 3627.9 | 3627.90 | 60.06 | 0.003 |
| Eluent concentration (mol/L) | 1 | 442.3 | 442.34 | 7.32 | 0.004 |
| Elution time (min) | 1 | 760.6 | 760.60 | 12.59 | 0.150 |
| Square | 4 | 3109.0 | 77.24 | 12.87 | 0.030 |
| Sonication time (min)*Sonication time (min) | 1 | 1361.0 | 1361.03 | 22.53 | 0.050 |
| Eluent concentration (mol/L) * Eluent concentration (mol/L) | 1 | 1458.8 | 1458.84 | 24.15 | 0.040 |
| pH*pH | 1 | 756.4 | 756.38 | 12.52 | 0.050 |
| Elution time (min)*Elution time (min) | 1 | 7.1 | 7.12 | 0.12 | 0.060 |
| 2-Way interaction | 6 | 183.9 | 30.65 | 0.51 | 0.010 |
| Sonication time (min)*pH | 1 | 39.7 | 39.74 | 0.66 | 0.050 |
| Sonication time (min)*Eluent concentration (mol/L) | 1 | 9.2 | 9.22 | 0.15 | 0.005 |
| Sonication time (mol/L) *Elution time (min) | 1 | 22.4 | 22.37 | 0.37 | 0.010 |
| pH*Eluent concentration (mol/L) | 1 | 105.1 | 105.09 | 1.74 | 0.060 |
| Error | 16 | 5.0 | 5.04 | 0.08 |  |
| Lack of fit | 10 | 827.8 | 82.78 | 0.34 |  |
| Pure error | 6 | 138.6 | 23.09 | 3.58 |  |
| Total | 30 | 10018.1 |  |  |  |

**Table S10:** Default weights used in AGREEprep.

| **Criteria** | **Criteria description** | **Default weights** |
| --- | --- | --- |
| 1 | Favor *in situ* sample preparation placement | 1 |
| 2 | Use safer solvents and reagents | 5 |
| 3 | Target sustainable, reusable, and renewable materials | 2 |
| 4 | Minimize waste | 4 |
| 5 | Minimize sample, chemical, and material amount | 2 |
| 6 | Maximize sample throughput | 3 |
| 7 | Integrate steps and promote automation | 2 |
| 8 | Minimize energy consumption | 4 |
| 9 | Choose the greenness possible post-sample preparation  configuration for analysis | 2 |
| 10 | Ensure safe procedures for the operator | 3 |

**Table S11:** Effect of various interfering cations on the preconcentration and determination of total Hg using online UA-m-DSPME method: Concentration of interfering ion = 100 µg L^-1^.

| **Cation** | **Recovery (%)** |
| --- | --- |
| Ag (I) | 97±0.1 |
| Cd (II) | 95.9±0.01 |
| Co (II) | 98.5±0.3 |
| Fe (II) | 99±0.2 |
| Pb (II) | 95±0.03 |
| Zn (II) | 98.9±0.09 |


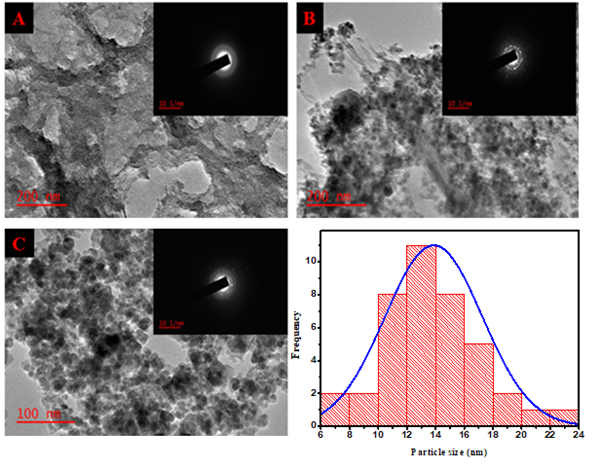


D

**Figure S1:** Transmission electron microscopy for (a) GO, (b) Fe_3_O_4_/GO, (c) Fe_3_O_4_/GO-Au and (d) particle side distribution of Fe_3_O_4_/GO-Au.

**Figure S2:** Recoveries (%) for (a) HCl, (b) HCl+ Au, and (c) HCl+ thiourea.


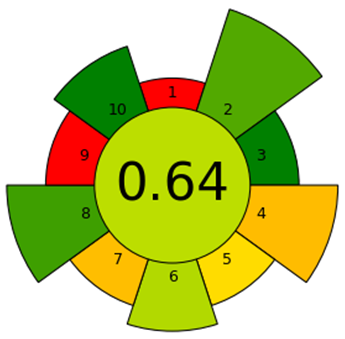


**Figure S3:** Pictogram for UA-m-SPME procedure.

**Figure S4:** Comparative analysis of the adsorbents GO, Fe_3_O_4_/GO, and Fe_3_O_4_/GO-Au for the total mercury measurement in fuel oils.


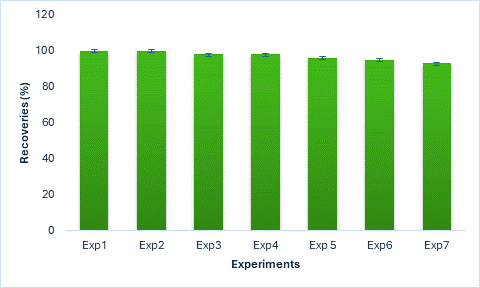


**Figure S5:** Reusability of the Fe_3_O_4_/GO-Au core−shell nanocomposite for adsorption of Hg in fuel matrices.

**Figure S6:** Magnetic hysteresis loop for reused Fe_3_O_4_-GO-Au nanocomposite.
